# Comparative Efficacy of Topical Metronidazole and Glyceryl Trinitrate Versus Topical Glyceryl Trinitrate Alone in the Treatment of Acute Anal Fissure: A Randomized Clinical Trial

**DOI:** 10.7759/cureus.31812

**Published:** 2022-11-23

**Authors:** M Hasaan Shahid, Sidra Javed, Saryia Javed, Anwar Zeb Khan, Adeel Kaiser, Reda H Mithany

**Affiliations:** 1 Surgery, Postgraduate Medical Institute, Lahore, PAK; 2 General Surgery, District Headquarter Hospital (DHQ) Hospital Okara, Okara, PAK; 3 General Surgery, Queen Elizabeth University Hospital, Glasgow, GBR; 4 Surgery, Lahore General Hospital, Lahore, PAK; 5 Laparoscopic Colorectal Surgery, Northampton General Hospital, Northampton, GBR

**Keywords:** proctological surgery, glyceryl trinitrate, anal fissure, metronidazole, colorectal disease, pr bleeding

## Abstract

Background and objective

An anal fissure is a longitudinal, oval lesion in the anal canal. In over 90% of instances, the anal fissures are located posterior to the midline and produce discomfort upon defecation and/or bleeding owing to spasms of the internal anal sphincter that leads to ischemia. This research aimed to determine if topical metronidazole treatment when combined with glyceryl trinitrate 0.2% (GTN), is more successful than GTN alone in reducing the time for an acute anal fissure to heal.

Material and methods

This study was a single-blinded, randomized controlled trial conducted at the DHQ Hospital Okara from January 2022 to August 2022. Patients of both genders, aged 18 to 70 years, with acute anal fissures, were included. One hundred forty patients who satisfied the inclusion criteria were randomized through the lottery technique and were divided into two groups (70 in each group). Group A contained patients who got metronidazole combination with GTN, while in Group B, patients treated with GTN alone without metronidazole. The primary endpoint was fissure healing, confirmed as finding a scar where the fissure was. While the secondary endpoint was maximum pain on defecation assessed by the Visual Analogue Scale (VAS). Statistical analysis was performed using Statistical Package for Social Sciences (SPSS) v24. Chi-Square and Fisher’s Exact tests were done for statistical analysis, and p < 0.05 was considered significant.

Results

Three patients lost the follow-up. Out of the remaining 137, 70 (51.1%) patients were male. The patient’s ages ranged from 22 to 68 years, with a mean age of 39.18 ± 11.52. One hundred twenty six (92%) complained of pain on defecation with a mean VAS of 6.01 ± 2.35. 80 (58.4%) patients complained of perianal itching, while 25 (18.2%) patients complained of bleeding on defecation.

On week 1 follow-up, in group A out of 69 patients, 27 (39.1%) had complete healing, 38 (55.1%) had partial healing, while in group B out of 68 patients, one (1.4%) had complete healing, 43 (63.2%) had partial healing (p = < 0.001, significant).

On week 3 follow-up, in group A out of 69 patients, 47 (68.1%) had complete healing, and 22 (31.8%) had partial healing, while in group B out of 68 patients, 16 (23.5%) had complete healing, 49 (72%) had partial healing (p = < 0.001, significant). Mean VAS score of group A was 0.61 ± 1.38 while that of group B was 2.57 ± 2.50 (p = < 0.001, significant).

Conclusion

Using topical metronidazole as an addition to standard therapy may reduce the chronicity of acute anal fissures and prevent surgical treatments with high rates of complications.

## Introduction

Lockhart-Mummery first defined the anal fissure in 1934 as a break in the mucosa ranging from the anal margin to the dentate line [1]. An anal fissure is a longitudinal, oval lesion in the anal canal. In over 90% of instances, the anal fissures are located posterior to the midline and produce discomfort upon defecation and/or bleeding owing to spasms of the internal anal sphincter (IAS) that leads to ischemia [2].

It is believed that the pathophysiology of anal fissures is connected to injury caused by passing hard faeces or persistent diarrhoea. A break in the anoderm induces contractions of the IAS, resulting in discomfort, further tearing, and a decrease in the anoderm's blood flow. The discomfort, spasm, and ischemia contribute to a persistent anal fissure from a poorly healed lesion [3].

Whereas most of the idiopathic fissures are situated in the posterior midline, a laterally positioned fissure should alarm the clinician to other probable underlying illnesses such as Crohn's disease, syphilis, anal cancer, acquired immune deficiency syndrome (AIDS), and tuberculosis [4].

Acute anal fissures last shorter than six weeks. They are superficial, limited to anoderm, and have sharp, fresh mucosal margins with granulation tissue at the base [5]. Chronic anal fissures (CAF) are characterized as resistant to medical treatment after six weeks. CAF is characterized morphologically by the presence of IAS fibres at the base of the fissure, with indurated margins, sentinel pile, and hypertrophied anal papilla [6].

Essential to treating anal fissures is maintaining soft, regular, and easily passed faeces. In the short term, bulk-forming or osmotic laxatives (such as ispaghula husk or lactulose, respectively) can promote good bowel movements; however, a high-fibre diet must be maintained to avoid recurrence. Effective pain management is also essential. This can be accomplished with common analgesics like paracetamol or ibuprofen. It has been demonstrated that topical anaesthetics, such as lidocaine 5% ointment, are beneficial for short-term usage. A rectal ointment containing 0.2% or 0.4% glyceryl trinitrate (GTN) can be recommended to promote the healing of anal fissures [7].

Injections of botulinum toxin are regarded as a successful therapy technique. Regarding the surgical treatment options for chronic fissures, lateral internal sphincterotomy (LIS) seems to have a cure rate of up to 96% and a recurrence rate of 2.3%-3% [8]. Since defects first induced by mechanical damage are in regular touch with faecal bacteria, they cannot be sterile and will ultimately develop an infection. Alteration in the microbial-tissue combination might result in nonspecific inflammation of the mucosa, resulting in the loss of elasticity and flexibility of the anoderm, which makes it susceptible to rupture. Via proinflammatory cytokines or microbial metabolites that restrict the regrowth of the mucosal membrane, they can also impede the healing process and turn acute wounds into chronic ulcers. As well as spasms of the anal sphincter and local ischemia, the status of the microbiota in the rectum, anal canal, and anal fissure itself strongly affects the recovery process of anal fissures [9].

Few studies with a small sample size have been done utilizing topical metronidazole; however, in the literature, comparison with GTN is minimal. Hence, the purpose of this study was to determine whether the use of topical metronidazole treatment could speed up the healing process of acute anal fissure in comparison to the use of GTN 0.2%, which has been used for years in the treatment of acute anal fissure and is considered to be more effective than the use of GTN alone.

## Materials and methods

This study was conducted at the District Headquarter (DHQ) Hospital Okara from January 2022 to August 2022 and was approved by the Hospital Ethics Committee (Institutional Review Board DHQ Hospital Okara, IRB No. 535-36/DHQ). This clinical trial is registered at The Registry for International Development Impact Evaluations (RIDIE) (registration no. RIDIE-STUDY-ID-6375ee39be092). Informed consent was obtained from all patients to be included in the study after explaining to them about the research. Patients of both genders, aged 18 to 70 years, with acute anal fissures, were included. An acute fissure was identified by the symptom's appearance duration the first time (present for less than six weeks). Exclusion criteria were CAF (symptoms present for more than six weeks) and the presence of anal sphincter fibrosis. This study was a single-blinded randomized controlled trial, and patients have not disclosed the exact treatment they were getting; however, they were aware of both therapies.

A total of 140 patients who satisfied the inclusion criteria were randomized through the lottery technique and were divided into two groups (70 in each group). Group A contained patients who got a metronidazole combination along with GTN; in Group B, patients were treated with GTN without metronidazole. The demographic details (age, gender, duration of symptoms, pain on defecation, pretreatment VAS score, bleeding and difficulty on defecation and peri-anal itching) were collected.

Patients in both groups were encouraged to prevent hard stools by dietary measures and were prescribed lactulose in syrup form 30mL thrice a day. Baseline assessment included recording the duration of symptoms, maximum severity of pain on defecation according to a visual analogue scale (VAS), and fissure healing. After the commencement of treatment, each patient was called for follow-up at week 1, week 3 and week 6. After complete healing of the ulcer, treatment was stopped. The primary endpoint was fissure healing, confirmed as finding a scar where the fissure was. While the secondary endpoint was maximum pain on defecation assessed by VAS.

Statistical analysis was performed using Statistical Package for Social Sciences v24. T-test, Chi-Square and Fisher’s Exact tests were done for statistical analysis, and p < 0.05 was regarded as significant.

## Results

Out of 140 patients (70 in each group), three patients lost the follow-up (one from Group A and two from Group B), hence excluded from the study. Out of the rest of 137, 70 (51.1%) patients were male while 67 (48.9%) were female (p = 0.5; not significant). Patients’ ages ranged from 22 to 68 years with a mean age of 39.18 ± 11.52. The mean age of patients in group A was 38.71 ± 10.78 while in group B it was 39.65 ± 12.28 (p = 0.634; not significant). Table 1 demonstrates the demographic characteristics of the patients.

**Table 1:** The demographic characteristics of the patients

	Group A (n=69)	Group B (n=68)	p-value
Gender distribution			
Males	41 (59.4%)	29 (42.6%)	0.5 (Not significant)
Females	28 (40.5%)	39 (57.3%)	
Age distribution (years)			
Min.	23	22	0.634 (Not significant)
Max.	67	68	
Mean ± SD	38.71 ± 10.78	39.65 ± 12.28	

**Figure 1:**
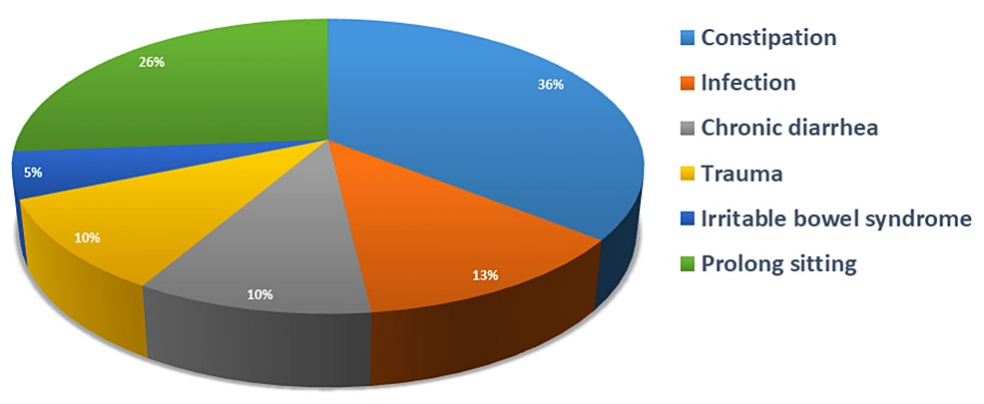
Etiological factors of anal fissure

The mean duration of symptoms in group A was 3.12 ± 1.17 weeks while that of group B was 2.79 ± 2.79 weeks (p = 0.115; not significant) (Table 2). Etiological factors are demonstrated in Figure 1. Out of 137, 126 (92%) complained of pain on defecation while 11 (8%) experienced no pain on defecation (p = 0.772; not significant) with mean VAS 6.01 ± 2.35 (p = 0.712; not significant). Eighty (58.4%) patients complained of perianal itching while 57 (41.6%) experienced no perianal itching. One hundred fourteen (83.2%) patients complained of difficulty in defecation while 23 (16.8%) experienced no difficulty. Twenty-five (18.2%) patients complained of bleeding on defecation while 112 (81%) did not have bleeding on defecation. Duration of bleeding varied from one to three weeks with a mean duration of 1.25 ± 0.45 in group A and 1.46 ± 0.77 in group B (p = 0.526; not significant) (Table 2).

**Table 2:** Symptomatic analysis before treatment

	Group A (n=69)	Group B (n=68)	p-value
Duration of symptoms (Weeks)			
Mean ± SD	3.12 ± 1.17	2.99 ± 2.79	0.115 (Not significant)
Pain on defecation			
Yes	63 (91.3%)	63 (92.6%)	0.772 (Not significant)
No	6 (8.6%)	5 (7.3%)	
Mean VAS score	6.04 ± 2.44	5.99 ± 2.28	0.712 (Not significant)
Difficulty in defecation			
Yes	58 (84%)	56 (82.3%)	0.789 (Not significant)
No	11 (15.9%)	12 (17.6%)	
Perianal itching			
Yes	41 (59.4%)	39 (57.3%)	0.471 (Not significant)
No	28 (40.5%)	29 (42.6%)	
Bleeding on defecation			
Yes	12 (14.3%)	13 (19.1%)	0.794 (Not significant)
No	57 (82.6%)	55 (80.8%)	
Duration of bleeding (weeks) Mean ± SD	1.25 ± 0.452	1.46 ± 0.776	0.526 (Not significant)

After the commencement of treatment, each patient was called for follow-up at end of week 1, week 3 and week 6. After complete healing of the ulcer, treatment was stopped.

On week 1 follow-up, in group A out of 69 patients, 27 (39.1%) had complete healing, 38 (55.1%) had partial healing while four (5.8%) had no healing while in group B out of 68 patients, one (1.4%) had complete healing, 43 (63.2%) had partial healing while 24 (35.2%) had no healing (p = < 0.001, significant). The mean VAS score of group A was 2.45 ± 2.16 while that of group B was 5.15 ± 1.62 (p = < 0.001, significant) (Table 3).

**Table 3:** Follow-up at the end of week 1 Visual Analogue Scale (VAS)

	Group A (n=69)	Group B (n=68)	p-value
Wound status			
Complete healing	27 (39.1%)	1 (1.4%)	< 0.001 Significant
Partial healing	38 (55.1%)	43 (63.2%)	
No healing	4 (5.8%)	24 (35.2%)	
VAS Score			
Min.	0	0	
Max.	7	8	
Mean ± SD	2.45 ± 2.16	5.15 ± 1.62	< 0.001 Significant

On week 3 follow-up, in group A out of 69 patients, 47 (68.1%) had complete healing and 22 (31.8%) had partial healing while in group B out of 68 patients, 16 (23.5%) had complete healing, 49 (72%) had partial healing while three (4.4%) had no healing (p = < 0.001, significant). The mean VAS score of group A was 0.61 ± 1.38 while that of group B was 2.57 ± 2.50 (p = < 0.001, significant) (Table 4).

**Table 4:** Follow-up at the end of week 3 Visual Analogue Scale (VAS)

	Group A (n=69)	Group B (n=68)	p-value
Wound status			
Complete healing	47 (68.1%)	16 (23.5%)	< 0.001 Significant
Partial healing	22 (31.8%)	49 (72%)	
No healing	0 (0%)	3 (4.4%)	
VAS Score			
Min.	0	0	
Max.	5	9	
Mean ± SD	0.61 ± 1.38	2.57 ± 2.50	< 0.001 Significant

On week 6 follow-up, in group A out of 69 patients, 67 (97.1%) had complete healing and two (2.8%) had partial healing while in group B out of 68 patients, 58 (85.2%) had complete healing and 10 (14.7%) had partial healing (p = 0.014, significant). The mean VAS score of group A was 0.07 ± 0.431 while that of group B was 0.38 ± 1.07 (p = 0.159, not significant) (Table 5).

**Table 5:** Follow-up at the end of week 6 Visual Analogue Scale (VAS)

	Group A (n=69)	Group B (n=68)	p-value
Wound status			
Complete healing	67 (97.1%)	58 (85.2%)	0.014 Significant
Partial healing	2 (2.8%)	10 (14.7%)	
No healing	0 (0%)	0 (0%)	
VAS Score			
Min.	0	0	
Max.	3	4	
Mean ± SD	0.07 ± 0.431	0.38 ± 1.07	0.159 Not significant

There was a recurrence of symptoms in one (1.4%) patient of group B while no recurrence was seen in group A. No local complications were seen in any patients in either group; however, eight (5.8%) patients complained of headaches, among them three (4.3%) were from group A while five (7.3%) were from group B. As a rescue dose for these headaches, paracetamol was prescribed and was effective.

## Discussion

In our study, it was shown that the use of GTN and topical metronidazole for the treatment of acute anal fissure, compared to the use of topical GTN alone, reduced the severity of pain in a very short period and both shortened the healing time of the fissure and increased the healing rate.

Typical presenting symptoms in acute anal fissures are pain on defecation and bleeding. Pain is usually sharp and aggravated during defecation. Sometimes painful defecation may be accompanied by bleeding. Similar to the literature, all of our patients had complaints of pain at defecation, and pain scores were found to be very high before starting treatment. In addition, defecation was accompanied by bleeding in 18% of the patients. These results are comparable to the findings of the study of Acar et al. in which pain due to anal fissure was present in 97% of patients with anal fissures; however, in contrast, the bleeding rate was also high in that study as compared to this study, may be due to chronicity of the disease [10].

Constipation is a major risk factor in the development of anal fissures, and fissures can further complicate due to itching in the perianal region. In another study conducted by Turkish researchers, the incidence of both of these conditions was significant and comparable to the results of our study [11].

Our results showed that antibiotic therapy with metronidazole and conventional therapy has early healing and pain relief in acute anal fissures. A study by Grekova et al. shared similar results and concluded that topical antibiotic therapy could be effective in patients with CAFs if the bacteria are correctly identified [9].

A study done by Karapolat concluded that in conjunction with traditional medical therapies, topical antimicrobial therapy with metronidazole is an efficient, simple-to-use, safe, quick, and painless technique that reduces anal fissure symptoms and accelerates healing. With this promising treatment, cases with acute anal fissures can be prevented from becoming chronic, and patients can be saved from being subject to future surgical interventions that involve high complication rates [12].

Research conducted in Georgia suggested that the notion behind using topical metronidazole to treat pain in the perineum was to make the drug more bioavailable. In theory, the anti-inflammatory and antimicrobial effects would be stronger in tissues than with the same dose taken by mouth. This evidence supports our study in that the mean VAS score in group A after initiating treatment was significantly less than in the other group [13].

A study done by Mert to compare the effects of metronidazole in combination with diltiazem demonstrated comparable results in acute anal fissures. Their trial showed healing in 66% of patients by the fourth week in the metronidazole group. It concluded that with a combination of topical metronidazole to standard therapy, the rapid decrease of acute anal fissure-related discomfort, the shortened healing time of the fissure, and the high healing rate show that infection plays a significant role in the aetiology of anal fissures. Using topical metronidazole as an addition to standard therapy may reduce the chronicity of acute anal fissures and prevent surgical treatments with high rates of complications [14]. 

In this study, 92% of patients experienced perianal pain with VAS 6.01 ± 2.35, which significantly decreased by the use of topical therapy in both groups; however, VAS was significantly less in patients who underwent metronidazole treatment. Comparable results were also seen in the study done by Saba et al. where VAS in the metronidazole group dropped from 7.4 ± 0.9 to 1.86 ± 0.48 over six weeks duration [15].

Nitroglycerine-related headaches were the only adverse effect faced by the patients in our study. This was also observed by the systematic review conducted by Alnasser et al. in which multiple studies claimed postural dizziness and headache due to nitrates use [16]. However, as a rescue dose, paracetamol was good enough to manage these headaches.

Limitations

Our study was limited to patients with acute anal fissures with less sample size. The paediatric population was also not included in this study as well. To evaluate this regime further, studies on larger scales with both acute and chronic conditions should be done, and effects on the paediatric population should be analysed.

## Conclusions

With the addition of topical metronidazole to standard therapy, the rapid decrease of acute anal fissure-related discomfort, the shortened healing time of the fissure, and the high healing rate show that infection plays a significant role in the aetiology of anal fissures. Using topical metronidazole as an addition to standard therapy may reduce the chronicity of acute anal fissures and prevent surgical treatments with high rates of complications.
